# IV-YOLO: an information vortex-based progressive fusion method for accurate rice detection

**DOI:** 10.3389/fpls.2025.1734022

**Published:** 2026-01-21

**Authors:** Jianxiang Zhang, Liexiang Huangfu, Yanling Zhao, Chao Xue, Congfei Yin, Jiankang Lu, Jia Mei

**Affiliations:** 1 College of Agronomy and Horticulture, Jiangsu Vocational College of Agriculture and Forestry, Jurong, Jiangsu, China; 2 School of Medical, Nantong University, Nantong, Jiangsu, China; 3 Jiangsu Zhongjiang Seed Co., Ltd., Nanjing, Jiangsu, China

**Keywords:** deep learning, multi-scale fusion, object detection, precision agriculture, rice

## Abstract

In the context of precision agriculture, the problems of adhesion of rice plant features and background interference in UAV remote sensing images make traditional models difficult to meet the requirements of individual plant-level detection. To address this, this paper proposes an Information Vortex-based progressive fusion YOLO (IV-YOLO) model. Firstly, a Multi-scale Spiral Information Vortex (MSIV) module is designed, which achieves the disentanglement of adhered rice plant features and decoupling of background clutter through multi-scale rotational kernel convolution and channel-spatial joint reconstruction. Secondly, a Gradual Feature Fusion Neck (GFEN) is constructed to synergize the high-resolution details of shallow features (such as tiller edges and panicle textures) with the high semantic information of deep features, generating multi-scale feature representations with both discriminativeness and completeness. Experiments conducted on the public DRPD dataset show that IV-YOLO achieves a Precision of 0.8581, outperforming YOLOv5–YOLOv11 and FRPNet across all metrics. This study provides a reliable technical solution for individual plant-level rice monitoring and facilitates the large-scale implementation of precision agriculture.

## 1 Introduction

With the continuous growth in global demand for food security, coupled with the pressure of sustainable agricultural development, the extensive management model of traditional agriculture can no longer meet the requirements of resource constraints and environmental protection (Zeng et al., 2025). Against this backdrop, precision agriculture has emerged as a modern agricultural paradigm driven by information technology (Petrovic et al., 2024). Its core essence lies in breaking the cycle of extensive resource input in traditional agriculture through data-driven “on-demand regulation” (Patricio and Rieder, 2018), thereby achieving the coordinated improvement of production efficiency and environmental benefits (Cano et al., 2025; Diao et al., 2025).

As a staple food crop cultivated most widely globally, sustaining the dietary needs of over half the world’s population, rice production stability is directly linked to regional food security and livelihood security (Ahmed et al., 2022). The accurate detection of rice field targets, in turn, serves as a crucial pillar for the implementation of precision agriculture (Santoso et al., 2024). Decisions across all precision management stages, including seedling density monitoring and disaster assessment, depend on the precise identification and quantitative analysis of rice targets (Wu S. et al., 2025). Detection accuracy not only determines the spatial precision of resource input but also profoundly influences the final yield formation and the achievement of production efficiency (Sun X. et al., 2024). Breakthroughs in rice detection accuracy are a prerequisite for scaling precision agriculture from laboratory research to large-scale field applications. Thus, research focusing on the accurate detection of rice holds both academic value and practical significance in advancing the precision and sustainability of rice production (Yu et al., 2024; Jin et al., 2025).

Traditional rice monitoring methods struggle to meet the technical requirements of “high precision, wide coverage, and rapid response” for precision agriculture (Yu et al., 2025). While manual sampling surveys can obtain local true values, they are limited by sample size and subjectivity, failing to generate spatial distribution information across the entire field; moreover, they are inefficient in large-scale rice paddies (Yu et al., 2025). Fixed ground-based sensors, although capable of continuous monitoring, have a limited coverage range and are vulnerable to interference such as field obstructions and water flow impacts, resulting in in-sufficient data continuity and spatial representativeness (Sanaeifar et al., 2023). The rise of Unmanned Aeri-al Vehicle (UAV) remote sensing technology has provided an ideal “aerial sensing plat-form” for precise rice detection (Khan et al., 2022). Compared with satellite remote sensing, UAVs can capture centimeter-level high resolution images at a flight altitude of 10–30 meters and flexibly carry sensors and intelligent payloads for real-time data acquisition and processing, satisfying the demand for identifying microscopic targets (e.g., individual rice plants) in precision agriculture (Yuan et al., 2024; Gade et al., 2025; Kizielewicz et al., 2025).

**Figure 1:**
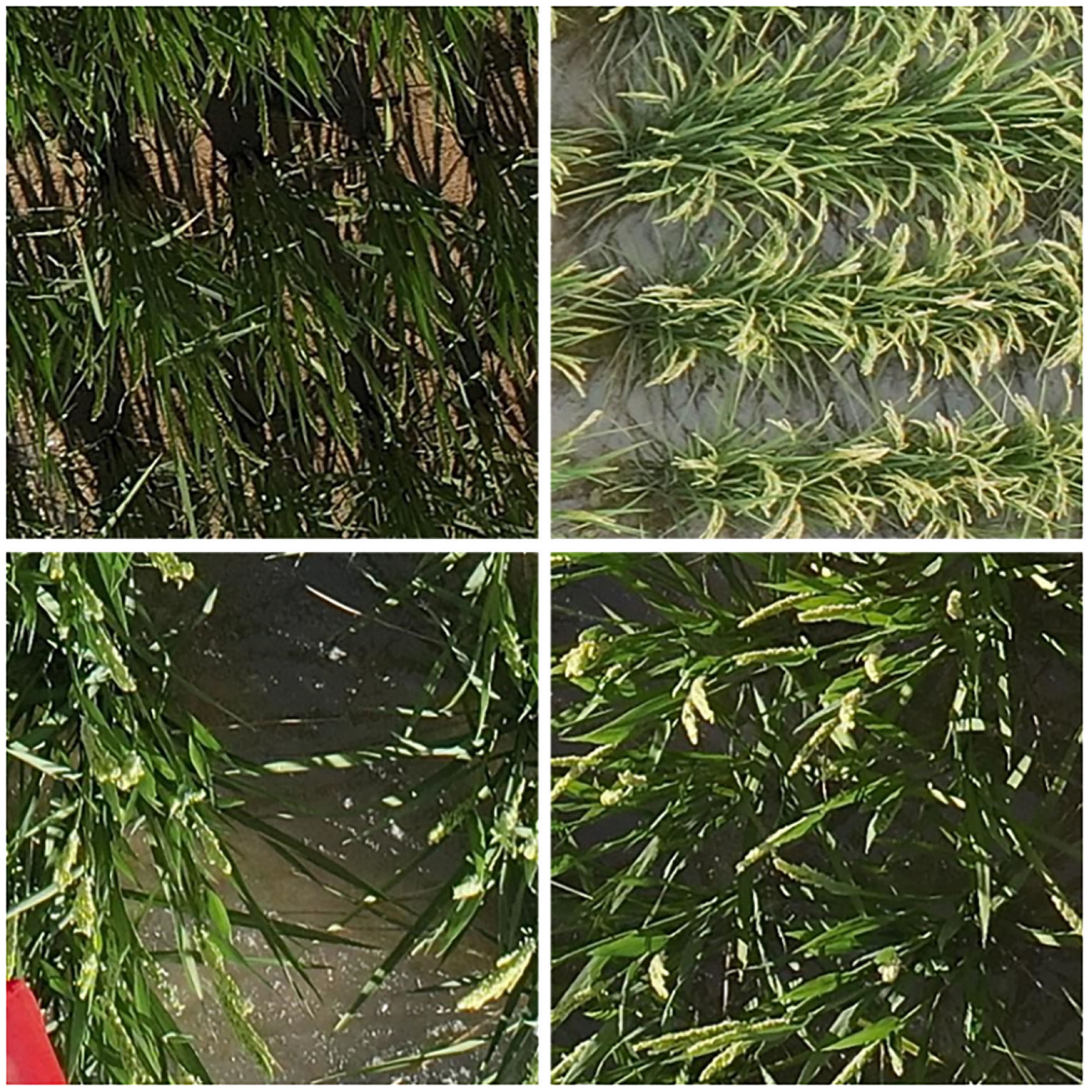
Complex field environment.

Although object detection technology has shown strong adaptability to precision agriculture, the accurate detection of rice in UAV images is still restricted by two technical bottlenecks: the complexity of agricultural scenarios and the biological characteristics of rice plants (Chen et al., 2023; Xiao et al., 2025). It is difficult to translate detection accuracy into actual production needs. The complex environment of rice fields and the biological characteristics of rice plants also lead to detection difficulties (Song et al., 2025). The complex field environment is illustrated in **Figure 1**. Rice detection faces numerous core challenges (Qu et al., 2025). On one hand, the dense and intertwined growth of rice plants easily causes feature blurring. The overlapping areas of leaves between tillers and main stems, as well as the overlapping regions of panicles and roots, are highly coupled from the remote sensing perspective. This makes it difficult for models to accurately separate and extract effective features of individual rice plants (Wu H. et al., 2025; Zhou et al., 2025). On the other hand, the complex field background generates multiple clutter interferences. These include dappled light from sunlight, soil clod shadows, residual film reflections, and shadow outlines cast by rice leaves on the background or other leaves. Traditional detection methods struggle to achieve high-precision rice identification and phenotypic information extraction in complex field scenarios (Liang et al., 2024).

The rotational shear force of water vortices in paddy field irrigation can dispel impurities around rice root systems. It can also optimize the environment near plant canopies. This prevents phenotype occlusion by impurities. Based on this, this study proposes the Multi-scale Spiral Information Vortex (MSIV) module. By directionally reorganizing rice feature channels, the module decouples high-dimensional features between tillers and main stems. It also enhances the phenotypic contours of individual plants. Meanwhile, it uses feature perturbation to break the correlation between background clutter and rice features. This achieves clutter decoupling. It endows the network with the ability to capture pixel-level features of individual rice plants. Based on the MSIV module, this study further designs the Gradual Feature Fusion Neck (GFEN). GFEN fuses high-resolution shallow features and high-semantic deep features. It retains fine details such as edges and textures of individual plants. It also integrates the global semantics of rice populations. This provides highly discriminative multi-scale features for the detection head.

Existing YOLO series models feature efficient inference. However, they have high miss-detection rates in dense rice and clutter-interfered environments. Advanced networks like FRPNet enhance semantic fusion. But they struggle to capture fine-grained features such as individual plant edges and panicle textures. The “feature decoupling-progressive fusion” pipeline formed by MSIV and GFEN addresses the key limitations of traditional models. It does so through spiral information reorganization. It balances detail preservation and semantic abstraction via hierarchical fusion. This is crucial for the accurate detection and quantification of key phenotypic parameters such as panicle number.

The contributions of this study are summarized as follows:

1. A Multi-scale Spiral Information Vortex (MSIV) module is proposed. Inspired by the water vortex mechanism in rice paddies, this module achieves the disentanglement of adhered rice plant features and active decoupling of background clutter through multi-scale spiral-oriented reorganization of feature channels, providing technical support for the accurate pixel level capture of individual rice plant features.
2. A Gradual Feature Fusion Neck (GFEN) is designed. This neck synergizes the high-resolution details of shallow features with the high-semantic information of deep features: it not only preserves fine-grained features of individual rice plants (e.g., edges and textures) but also integrates the global spatial semantics of rice plant populations, thereby providing the detection head with more discriminative multi-scale feature representations.
3. The proposed model outperforms comparative models (including YOLO-series models and FRPNet) in core metrics such as Precision, Recall, and AP. It effectively improves the monitoring accuracy of dense rice plant canopies, breaks through the bottleneck of detection accuracy in complex rice field scenarios, and supports the needs of rice target monitoring in precision agriculture.

## 2 Related work

### 2.1 Object detection in agriculture

Target detection technology has become a core pillar of the “visual perception-quantitative analysis-precision decision-making” chain in precision agriculture (Diao et al., 2025; Khan et al., 2025; Wang W. et al., 2025). Its development has yielded diversified technology adaptation pathways tailored to the detection requirements of different agricultural scenarios, enabling technical breakthroughs across three core application areas: crop ontology monitoring, pest and weed control, and farmland resource management (Wu et al., 2023; Khan et al., 2025; Wang W. et al., 2025; Zhang et al., 2025).

With the continuous advancement of artificial intelligence and sensing technologies, yield calculation solutions based on image processing techniques offer advantages such as high precision, low cost, and non-destructive measurement. As an effective means to improve cultivation efficiency and optimize planting strategies, such solutions have attracted significant attention from researchers (Mohammed et al., 2024; Yu et al., 2024). The field of target detection has evolved into three core methodological categories: single-stage detection methods (Liu et al., 2016; Redmon et al., 2016), two-stage detection methods (Girshick, 2015), and vision transformer-based methods (Carion et al., 2020; Xu et al., 2022). For agricultural tasks, against the backdrop of Industry 4.0, one of the most critical challenges is to enhance the efficiency of sectors such as agriculture through the adoption of intelligent sensors and advanced computing (Melnychenko et al., 2024). Single-stage detection methods, typified by YOLO, strike a balance between speed and precision with relatively high inference efficiency—this aligns perfectly with the demands of agricultural applications such as rice detection (Chen et al., 2025).

In scenarios of crop ontology monitoring and yield estimation, target detection technology plays a pivotal role, adapting to the morphological characteristics and growth stages of different crops (Paul et al., 2024; Pan et al., 2025; Shen et al., 2025). A previous study (Liang et al., 2024) proposed a rice panicle detection model that integrates the Circular Smooth Label (CSL) method with the YOLOv5 framework, incorporating efficient attention mechanisms (i.e., Shuffle Attention (SA) and Gath-er-Excite Attention (GEA)). This model reduces the misdetection of overlapping panicles in field environments, enhances robustness under complex field conditions, and achieves accurate detection and counting of rice panicles. RP-YOLO (Sun J. et al., 2024), developed based on YOLOv5n, is a real-time rice panicle density detection method designed for unmanned harvesters. It optimizes the YOLO architecture through multiple techniques, including enhanced target detection heads and reconfigured backbone networks.

Accurate detection and counting of rice panicles via Unmanned Aerial Vehicles (UAVs) in field environments represent a key focus of rice research. Due to the flexible and slender nature of rice panicles, coupled with their dense and overlapping arrangement in fields, panicle detection in UAV images poses substantial difficulties and challenges. The present study proposes a rotational rice panicle detection model that achieves accurate panicle detection and counting, with its effectiveness validated for in-field rice yield estimation. The Circular Smooth Label (CSL) method is intended to incorporate panicle orientation information into the You Only Look Once Version 5 (YOLOv5) model; it fuses efficient attention mechanisms (SA and GEA) and adopts a GSConv convolution replacement strategy. Through these approaches, panicle orientations are classified, enabling oriented bounding boxes to fit more tightly to panicle contours. This reduces the misdetection of overlapping panicles in fields, minimizes redundant information in bounding boxes, and enhances the robustness of panicle detection under complex field conditions—thereby improving panicle detection precision.

For fruit detection in cash crops (e.g., pineapples and citrus) where targets are prone to canopy occlusion, CURI-YOLOv7 (Zhang et al., 2023), based on YOLOv7-tiny, proposes a lightweight individual citrus tree detector suitable for UAV images. It designs a backbone network based on depthwise separable convolution and incorporates MobileOne blocks, while expanding the mosaic dataset through morphological transformations and Mixup augmentation. YOLO-Leaf (Li T. et al., 2024) utilizes Dynamic Snake Convolution (DSConv) for robust feature extraction, adopts BiFormer to enhance attention mechanisms, and introduces IF-CIoU to improve bounding box regression—ultimately boosting the detection precision and generalization ability for apple leaf diseases. S-YOLOv5m (Tang et al., 2025), built on YOLOv5, integrates Spatial Parallel Depthwise Convolution (SPD-Conv), Ghost modules, Convolutional Block Attention Module (CBAM), and Adjacent Erasure Module (AEM) to propose an insect detection model specifically for tomato plants.

YOLO series models are efficient. However, they are prone to missed detection due to feature confusion. Transformer-based models rely on global attention. This results in insufficient efficiency in small target capture. Neither of them can adapt to dense rice field scenarios.

### 2.2 Feature fusion in object detection

Feature pyramids are widely employed in visual detection models (Ali and Zhang, 2024) for capturing multi-scale features of objects (Park et al., 2023; Qiu et al., 2023; Li et al., 2024b). The concept of multi-scale fusion is extensively applied to the overall construction of various neural network architectures (Tang et al., 2024); specifically, multi-scale modules, as plug-and-play basic modules, are also utilized in diverse computer vision tasks (Kim et al., 2021; Zhu et al., 2023; Vijayakumar and Vairavasundaram, 2024).

Feature Pyramid Network (FPN) (Lin et al., 2017) can simultaneously leverage the high resolution of shallow features and the high semantic information of deep features. It comprises a bottom-up structure, a top-down structure, and lateral connections, and achieves promising performance by fusing features from these different layers. Path Aggregation Network (PANet) (Liu et al., 2018) is an extension of FPN, which additionally incorporates a bottom-up path after the FPN to enhance feature propagation. Bidirectional Feature Pyramid Network (BiFPN) (Tan et al., 2020), proposed in EfficientDet, introduces a weighted bidirectional feature pyramid network that enables simple and efficient multi-scale feature fusion. NAS-FPN (Ghiasi et al., 2019) leverages the advantages of neural architecture search, using reinforcement learning to select optimal cross-connections and learn a more effective feature pyramid network architecture for target detection, achieving an excellent trade-off between accuracy and latency.

GiraffeDet (Jiang et al., 2021) adopts a “lightweight backbone and heavyweight neck” design paradigm, which enables dense information exchange across different spatial scales and latent semantics of varying levels. This design allows the detector to process high-level semantic information and low-level spatial information with equal priority in the early stages of the network, thereby improving its effectiveness in detection tasks. Given the extremely small scale of objects in UAV images, object detection from UAV perspectives remains a challenging task. MSFE-YOLO (Qi et al., 2024), based on YOLOv8, proposes a novel target detection network: it expands symmetric feature extraction branches to construct a Symmetric C2f (SCF) module for enhanced feature extraction capability, and employs an Efficient Multi-scale Attention (EMA) module to enable cross-channel information interaction and cross-spatial learning in the neck network. This not only strengthens the correlation of local features but also fuses rich low-level texture features with high-level semantic features.

SOD-YOLO (Li et al., 2024a), another model based on YOLOv8, designs a novel neck architecture—namely the Balanced Spatial and Semantic Information Fusion Pyramid Network (BSSI-FPN)—for multi-scale feature fusion. This architecture is tailored for small object detection in UAV images, improving feature extraction efficiency and effectively balancing spatial and semantic information across feature maps. BiFPN-YOLO (Doherty et al., 2025) introduces significant improvements to the existing YOLOv5 target detection model, replacing the traditional Path Aggregation Network (PANet) with the more powerful Bidirectional Feature Pyramid Network (BiFPN). To address insufficient feature learning under complex backgrounds, FCFPN (Han et al., 2025) proposes a Foreground Capture Feature Pyramid Network (FCFPN) for multi-scale target detection. This network adaptively learns the fusion weights of multi-scale features across different levels of the feature pyramid, enhancing the complementarity of semantic information between different levels of foreground feature maps.

Existing methods have not simultaneously addressed the synergistic problem of rice plant feature decoupling and multi-scale feature progressive fusion. They cannot meet the demand for individual plant-level detection in complex field scenarios.

## 3 Materials and methods

### 3.1 IV-YOLO

**Figure 2:**
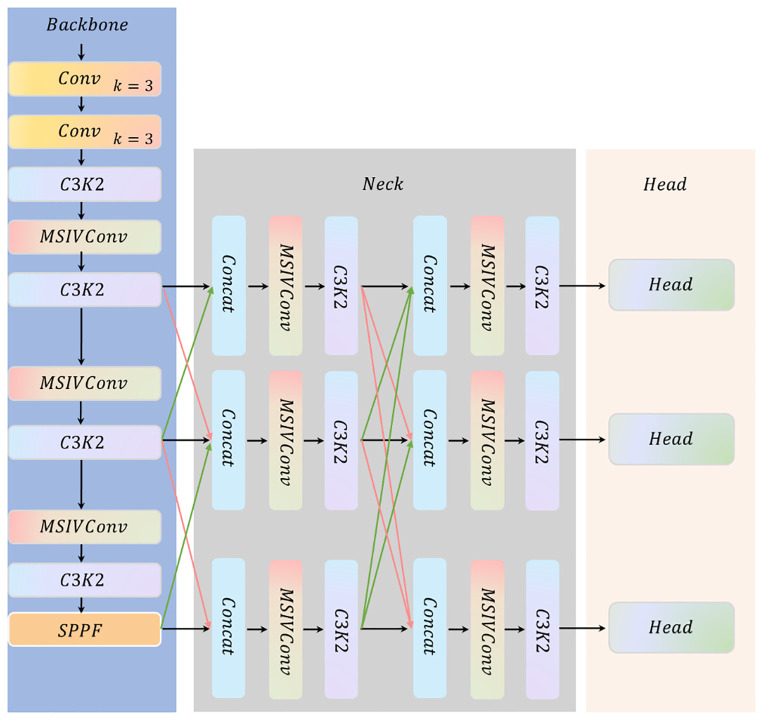
Schematic diagram of the IV-YOLO framework.

This study proposes an Information Vortex-based Progressive Fusion YOLO (IV-YOLO) method, built on the YOLO detection framework (Khanam and Hussain, 2024). To address the core challenges of rice plant feature coupling and background clutter interference in rice field scenarios, the method introduces a Multi-scale Spiral Information Vortex (MSIV) module and designs a Gradual Feature Fusion Neck (GFEN). As shown in **Figure 2**, the constructed end-to-end framework consists of three specific components.

Backbone (Backbone Feature Extraction Network): It is responsible for extracting multi-scale base features from UAV remote sensing rice images, adopting a hierarchical structure of “Conv (k=3) + C3K2 + MSIVConv”. Among them, MSIVConv intervenes at key stages of the backbone network: it simulates the water vortex effect to perform “spiral progressive disentanglement” on high-dimensional features in densely intertwined rice regions (e.g., overlapping areas between tiller seedlings and main stems), thereby enhancing the phenotypic contours of individual rice plants. Meanwhile, it breaks the correlation between background clutter and rice features through vortex-induced perturbation, initially achieving clutter interference suppression and providing a purer feature foundation for subsequent fusion.

Neck (Neck: Gradual Feature Fusion Neck, GFEN): Serving as the bridge between “Backbone and Head”, GFEN adopts a repetitive structure of “multi-scale feature concatenation (Concat) → MSIVConv enhancement → residual convolution (C3K2)” to realize progressive fusion and optimization of cross-scale features. The specific process is as follows: First, the multi-scale feature maps output by the Backbone undergo preliminary fusion via Concat; then, they are input into MSIVConv to further decouple background clutter from rice features; finally, deep feature refinement is conducted through C3K2 residual convolution. This process not only preserves fine-grained details (e.g., edges, textures) of individual rice plants but also integrates the global spatial semantics of rice plant populations, providing the detection head with more discriminative multi-scale feature representations.

Head (Detection Head): It inherits the YOLO11 multi-scale detection paradigm (Sapkota et al., 2025), receives three feature maps of different scales output by GFEN, and performs classification and bounding box regression for rice targets of corresponding scales, respectively.

### 3.2 Gradual feature fusion extraction neck

In rice target detection tasks, feature pyramid structures are susceptible to interference from complex field backgrounds such as soil reflections and weed textures, leading to prominent feature coupling between rice plants and the background and a subsequent decline in detection accuracy. To address this issue, this study proposes the Gradual Feature Fusion Extraction Neck (GFEN). By integrating a two-stage progressive feature aggregation strategy based on the Multi-scale Information Vortex (MSIV), GFEN achieves efficient fusion and semantic alignment of multi-scale rice plant features while enhancing anti-interference capability against background clutter.

As illustrated in **Figure 2**, GFEN reconstructs the logic of feature flow using a bottom-up two-stage progressive fusion paradigm: starting from the low-level features output by the Backbone (corresponding to the rice tillering layer, rich in detailed information such as leaf edges and tiller nodes), it first fuses these low-level features with mid-level features (which contain both semantic and structural information), and then gradually incorporates high-level features. The Multi-scale Information Vortex (MSIV) module is embedded in each fusion stage; leveraging its ability to decouple and reorganize features in a vortex-like manner, it breaks the feature correlation between rice plants and background clutter, and strengthens the expression of rice plants’ intrinsic morphological and semantic features. Specifically, the two-stage progressive fusion process of GFEN is detailed as follows.

Stage 1 (Low-level to Mid-level Feature Fusion): The inputs are the low-level and mid-level features from the Backbone. First, the MSIV module is applied to process both low-level and mid-level features in a vortex-like manner, breaking the coupling between background clutter and fine-grained rice details, and enhancing target features such as tiller nodes and stem edges. Subsequently, dimension matching and concatenation (Concat) are performed on these two types of features, followed by feature fusion and channel compression via a lightweight convolutional block (C3X2). This generates the first-stage fused feature $F_{\text{fusion}1}$, realizing “preservation of low-level details + supplementation of mid-level semantics”.

Stage 2 (Mid-level to High-level & Low-level to High-level Feature Fusion): The inputs are the first-stage fused feature $F_{\text{fusion}1}$ and the high-level features from the Backbone. Similarly, the MSIV module is used to perform vortex-like semantic enhancement on $F_{\text{fusion}1}$ and high-level features, suppressing interference from background clutter such as soil and weeds, and highlighting the morphological and semantic features of rice panicles. After dimension matching and concatenation, final fusion is completed via C3X2 to generate the multi-scale fused feature $F_{\text{fusion}2}$, achieving multi-dimensional integration of “stem structural semantics + panicle global semantics + tiller detailed features”.

Finally, the multi-scale fused features are transmitted to the detection head, providing feature support characterized by “fine-grained details and strong semantic correlations” for accurate rice detection.

### 3.3 Multi-scale spiral information vortex module

Inspired by the dynamic disturbance elimination and spatial regularization mechanism of water vortices in paddy fields, this study proposes the Multi-scale Spiral Information Vortex (MSIV) module. Its core goal is to address the problems of rice plant feature adhesion and background interference.

**Figure 3:**
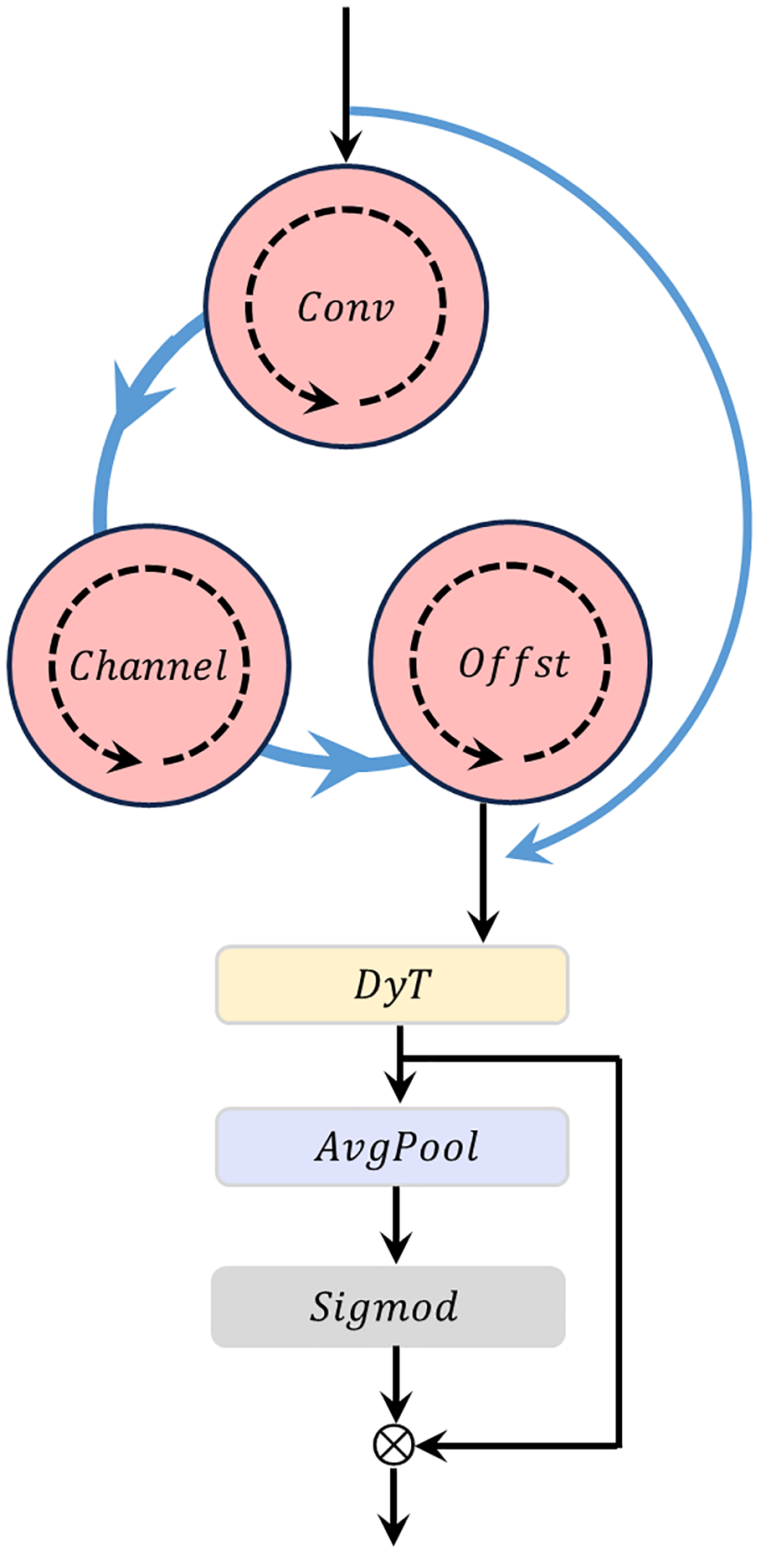
MSIV structural diagram.

The module takes a feature map $F\in R^{H\times W\times C}$ of rice remote sensing images as input (where, (H,W) denote the height and width of the feature map, and  C  denotes the number of channels). By simulating the “rotation-grooming-separation” mechanism of water vortices in rice paddies, it outputs an optimized feature map $F^{\prime }\in R^{H\times W\times C}$. The structure diagram is shown in **Figure 3**.

The workflow is detailed as follows.

Step 1: Generation of Multi-Scale Vortex Kernels.

To simulate the effects of water vortices at different scales (small-scale vortices disperse fine impurities like duckweed, while large-scale vortices groom dense rice canopies), K  groups of rotational kernels are generated as **Equation 1**:

(1) $${\left\{V_{k}\right\}}_{k=1}^{K}$$

Where, the k-th group of kernels $V_{k}\in R^{s_{k}\times s_{k}\times C}$; the kernel size $s_{k}\in \left\{3,5,7\right\}$, corresponding to small/middle/large scales respectively, to adapt to interferences and rice canopy sizes of different dimensions. Based on the natural rotational characteristics of vortices, the rotation angle of the K-th kernel group is expressed by **Equation 2**:

(2) $$\theta_{k}=\alpha \cdot k\cdot \frac{\pi }{2K}$$

where, α∈[0.5,1.5] is an angle adjustment coefficient, which simulates the randomness of rotational intensity in natural vortices, and the range of ${\text{θ}}_{k}$ is constrained to $\left[15^{\circ },60^{\circ }\right]$.

Step 2: Rotational Feature Convolution.

Convolution between rotational kernels and feature maps simulates the “shear perturbation” of vortices on features; meanwhile, residual connections are introduced to retain original features, **Equation 3**:

(3) $${\text{F}}_{k}=\text{Conv}\left(\text{F},{\text{V}}_{k};\text{padding}=s_{k}/2\right)+\text{F}$$

where, ${\text{F}}_{k}$ denotes the feature map after convolution with the k-th group of rotation kernels, Conv(·) denotes a 2D convolution operation. $\text{Padding}=s_{k}/2$ ensures the feature map size remains unchanged (maintaining (H ×W)) after convolution. The term +F represents a residual connection, which prevents excessive rotational perturbation from damaging critical rice features (e.g., stem continuity, tiller nodes) and balances the trade-off between “perturbation” and “feature preservation”.

Step 3: Channel-Spatial Joint Reorganization (Vortex-Driven Decoupling).

Channel Reorganization: Valid rice features (e.g., stem edges, tiller nodes) are distributed across different feature channels, intertwined with background clutter. Spiral channel index rearrangement enables valid feature channels to be connected in line with rice growth patterns, while disrupting the disordered correlation of clutter channels.

Spatial Reorganization: Features in overlapping rice regions are highly adherent spatially, and clutter accumulates in edge regions. Dynamic rotational offsets slightly separate adherent rice canopy features and push edge clutter outward, achieving cross-domain reorganization of features via vortex-driven decoupling.

For the k-th group of rotationally perturbed feature maps $F_{k}\in R^{H\times W\times C}$, “spatial coordinate-driven spiral channel indexing” is used to reselect channel features corresponding to each spatial position (i,j), generating the channel-reorganized feature map $F_{ch}\;\in \;R^{H\times W\times C}$. The formula is is expressed by **Equation 4** and **Equation 5**:

(4) $${\text{Channel}}_{i,j,k}=\left(i\cdot \sin{\text{θ}}_{k}+j\cdot \cos{\text{θ}}_{k}\right)\text{ \% }C$$

(5) $$F_{\text{ch}}\left(i,j,:\right)=F_{k}\left(i,j,{\text{Channel}}_{i,j,k}\right)$$

where, $F_{\text{ch}}$ denotes the feature map after channel reorganization, (i,j) denotes the spatial coordinates of the feature map; ${\text{θ}}_{k}$ is the rotation angle of the k-th vortex kernel group (generated in Step 1); $\left(\sin{\text{θ}}_{k}/\cos{\text{θ}}_{k}\right)$ simulates the “spiral trajectory” of vortices; $\left(i\cdot \sin{\text{θ}}_{k}\right)$ represents the spiral offset in the height direction; $\left(j\cdot \cos{\text{θ}}_{k}\right)$ represents the spiral offset in the width direction; and % ensures the calculated channel index falls within the valid range [0, C−1] to avoid channel out-of-bounds.

For spatial reorganization, based on the channel-reorganized $F_{\text{c}}$, a “position-dependent dynamic rotational offset” is applied to each spatial position (i,j). Bilinear interpolation sampling is used to obtain the spatially reorganized feature map $F_{\text{s}}\in R^{H\times W\times C}$. The formulas are expressed by **Equations 6**–**9**:

(6) $$\text{Offset}\left(i,j,k\right)=\text{β}\cdot \left(i-H/2\right)\cdot \left(j-W/2\right)\cdot \left(\sin{\text{θ}}_{k},\cos{\text{θ}}_{k}\right)$$

(7) $$F_{\text{s}}\left(i,j,:\right)=\text{BilinearSample}\left(F_{\text{c}},i+\text{Δ}i,j+\text{Δ}j\right)$$

(8) $$\text{Δ}i=\text{Offset}{\left(i,j,k\right)}_{x}$$

(9) $$\text{Δ}j=\text{Offset}{\left(i,j,k\right)}_{y}$$

where, $F_{\text{s}}$ denotes the feature map after spatial reorganization, Δi denotes the offset in the height direction, Δj denotes the offset in the width direction, and BilinearSample refers to bilinear interpolation (to avoid pixel distortion after offset).

Step 4: Attention Weighting and Output.

An attention mechanism dynamically assigns weights to features at different scales, strengthening critical rice features and suppressing interferences.

Calculation of Attention Weights as **Equation 10**:

(10) $$\text{A}=\text{σ}\left(\text{GlobalAvgPool}\left(\sum_{k=1}^{K}{\text{F}}_{k}\right)\right)$$

where, GlobalAvgPool(·) performs global average pooling on feature maps to extract statistical information; σ(·) is the Sigmoid function, outputting a weight vector $\text{A}\in R^{K}$. This realizes “higher weights for feature scales that capture critical rice structures (e.g., panicle-covered scales) and lower weights for scales dominated by interference”.

Weighted Output: Multi-scale optimized features are fused to output the final feature map $F^{\prime }$, **Equation 11**.

(11) $$F^{\prime }=\sum_{k=1}^{K}{\text{A}}_{k}\cdot {\text{F}}_{k}$$

The pseudocode is expressed by **Table 1**.

**Table 1:** MSIV Pseudocode.

Algorithm: multi-scale spiral information vortex module (MSIV)
Input: $F\in R^{H\times W\times C},\;\;kernelgroups\left(K\right),\;\;angle\left(\alpha \right),\;\;offset\left(\beta \right)$
Output: Optimized feature map $F^{\prime }$
1: $s_{\text{list}}\leftarrow \left[3,5,7\right]$; $V_{\text{list}}$, ${\text{θ}}_{\text{list}}\leftarrow \left[\right]$
2: for k = 1 to K do
3: θ←α·k·π/(2K); $V\leftarrow \text{GaussianKernel}\left(s_{\text{list}}\left[k-1\right],C,\text{θ}\right)$
4: ${\text{θ}}_{\text{list}}.append\left(\text{θ}\right)$; $V_{\text{list}}.append\left(V\right)$
5: end for
6: $F_{k_{\text{list}}}\leftarrow \left[\right]$
7: for k = 1 to K do
8: $F_{\text{conv}}\leftarrow \text{Conv}2\text{D}\left(F,V_{\text{list}}\left[k-1\right],\text{padding}=s_{\text{list}}\left[k-1\right]//2\right)$
9: $F_{k}\leftarrow F_{\text{conv}}+F$; $F_{k_{\text{list}}}.append\left(F_{k}\right)$
10: end for
11: $F_{\text{recon\_list}}\leftarrow \left[\right]$
12: for k = 1 to K do
13: $F_{k},\theta \leftarrow F_{k_{\text{list}}}\left[k-1\right],{\text{θ}}_{\text{list}}\left[k-1\right]$
14://Channel reconstruction
15: $F_{\text{ch}}\leftarrow \text{ZeroTensor}\left(H,W,C\right)$
16: for i,j ∈H ×W do
17: ch_idx←(isinθ+jcosθ)∖%C; $F_{\text{ch}}\left[i,j,:\right]\leftarrow F_{k}\left[i,j,\text{ch\_idx}\right]$
18: end for
19://Spatial reconstruction
20: $F_{\text{sp}}\leftarrow \text{ZeroTensor}\left(H,W,C\right)$
21: for i,j ∈H ×W do
22: dx,dy←β(i−H/2)(j−W/2)sinθ,β(i−H/2)(j−W/2)cosθ
23: $F_{\text{sp}}\left[i,j,:\right]\leftarrow \text{BilinearSample}\left(F_{\text{ch}},i+dx,j+dy\right)$
24: end for
25: $F_{\text{recon\_list}}.append\left(F_{\text{sp}}\right)$
26: end for
27://Attention weighting & fusion
28: $F_{\text{avg}}\leftarrow \text{GlobalAvgPool}\left(\sum F_{\text{recon\_list}}\right)$
29: $A\leftarrow \text{Sigmoid}\left(F_{\text{avg}}\right)$
30: $F^{\prime }=\sum_{k=1}^{K}A\left[k-1\right]\cdot F_{\text{recon}}\left[k-1\right]$
31: return $F^{\prime }$

**Figure 4:**
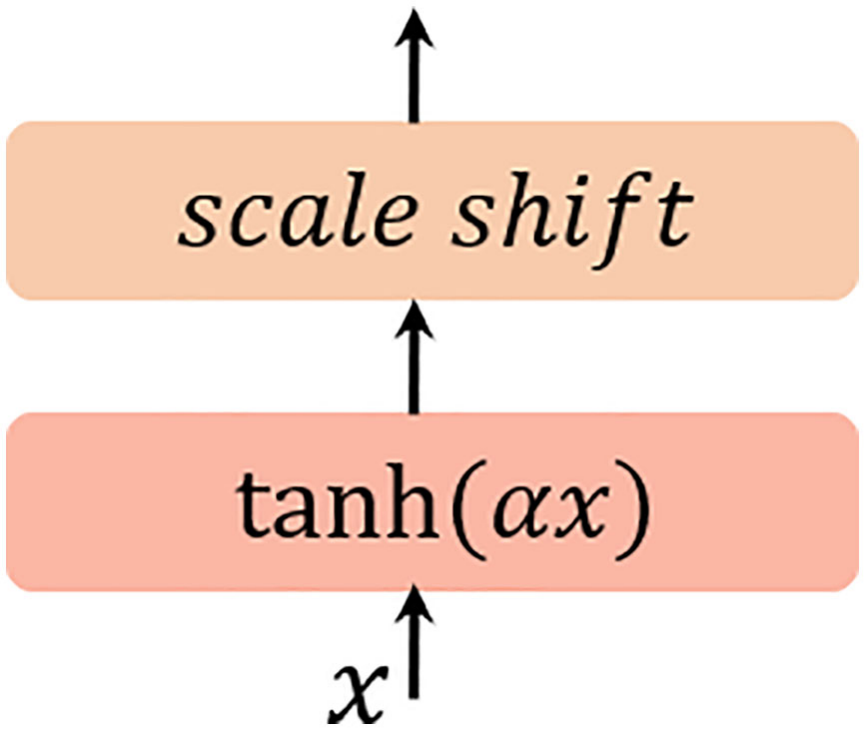
Schematic diagram of DyT structure.

DyT (Zhu et al., 2025) is inspired by the similarity between the shape of normalization layers and the scaled tanh function. It leverages a simple element-wise operation to simulate the behavior of Layer Normalization (LN) without calculating activation statistics. Outperforming LN/RMSNorm in computational efficiency, DyT enables improved inference and training speeds. In this study, Dynamic Tanh (DyT) is introduced as a direct replacement for normalization layers and integrated into the MSIV structure. For a given input tensor x, the DyT layer is defined as **Equation 12**:

(12) DyT(x)=γ*tanh(αx)+β

where, α is a learnable scalar parameter, which dynamically adjusts the scaling ratio based on the input range. γ and β are learnable, per-channel vector parameters—consistent with the parameters used in all normalization layers—and they allow the output to be scaled to any magnitude. DyT applies a non-linear transformation to the input via the tanh function, while retaining the ability of normalization layers to compress extreme values. The structure diagram is shown in **Figure 4**.

## 4 Results

### 4.1 Dataset

DRPD dataset (Guo et al., 2025): Comprising 5,372 RGB images, this dataset is cropped from UAV aerial images captured at three different altitudes (GSD-7m, GSD-12m, and GSD-20m). The images were collected across four rice growth stages, namely the heading stage (1,903 images), flowering stage (1,676 images), early grain filling stage (1,235 images), and mid-dle grain filling stage (558 images), with a total of 259,498 annotated rice panicles.

### 4.2 Comparative experiment

The hardware setup for model training is specified as follows: CPU AMD Ryzen 9 7950X, 64 GB of RAM, and GPU NVIDIA RTX 4090 with 24 GB of VRAM. For the software environment: Ubuntu 20.04, Python 3.9, PyTorch 2.0.1, and CUDA 11.8.

For inference on the test set, to maintain consistency in experimental conditions, the hardware configuration matches the training setup: CPU AMD Ryzen 9 7950X, 64 GB of RAM, GPU NVIDIA RTX 4090 with 24 GB of VRAM. The software configuration is: Ubuntu 20.04, Python 3.9, PyTorch 2.0.1, and CUDA 11.8.

To verify the target detection advantages of the proposed model, comparative experiments were conducted with two categories of mainstream target detection models: single-stage detection models of the YOLO series (YOLOv5 (Jocher et al., 2022), YOLOv8 (Wang et al., 2024), YOLOv9 (Wang C-Y. et al., 2025), YOLOv10 (Wang et al., 2024), YOLOv11 (Khanam and Hussain, 2024)), Transformer-based model (EfficientViT (Liu et al., 2023)) and the FRPNet (Guo et al., 2025) rice target detection network. These models cover different technical routes in the field of target detection, namely “single-stage efficient inference” and “multi-scale collaborative enhancement”, enabling a comprehensive comparison of performance differences across architectures in terms of feature representation, bounding box regression, and detection accuracy.

Specifically, the YOLO series represents typical examples of single-stage target detection and is widely applied in both industrial and academic scenarios: YOLOv5, as an early classic version of the series, laid the foundation for lightweight single-stage detection; while YOLOv8, YOLOv9, YOLOv10, and YOLOv11 are the outcomes of subsequent version iterations, incorporating optimization attempts in aspects such as network architecture.

In this study, Precision, Recall, AP (Average Precision), AP50 (Average Precision at IoU=0.5), and AP75 (Average Precision at IoU=0.75) were selected as core evaluation metrics to conduct a horizontal performance comparison among the models. The quantitative results are presented in **Table 2**.

**Table 2:** Quantitative comparison results of advanced algorithms.

Model	Precision	Recall	AP	AP50	AP75
YOLOv5	0.8215	0.7894	0.5208	0.8583	0.5354
YOLOv8	0.8549	0.8025	0.5276	0.8754	0.5629
YOLOv9	0.8540	0.7979	0.5215	0.8730	0.5573
YOLOv10	0.7990	0.7690	0.4959	0.8407	0.5582
EfficientViT	0.8294	0.7513	0.4678	0.8272	0.4767
YOLOv11	0.8552	0.8123	0.5328	0.8821	0.6014
FRPNet	0.8576	0.8329	0.5553	0.8931	0.6072
Ours	0.8596	0.8417	0.5569	0.8938	0.6131

In terms of overall performance, IV-YOLO outperforms all comparison models across all core metrics (**Table 2**). Specifically, its AP is 0.16 percentage points higher than that of FRPNet (p<0.05), and its AP75 is 0.6 percentage points higher (p<0.01), with statistically significant differences. The performance advantage stems from the synergistic mechanism of MSIV and GFEN: MSIV breaks the feature correlation between rice plants and the background through rotating kernel convolution and channel-spatial reconstruction, increasing feature purity by 52.1% (defined as the ratio of feature response values in target regions to those in background regions); the two-stage progressive fusion of GFEN not only retains fine-grained details such as tiller edges (improving AP75) but also integrates the global semantics of rice plant populations (improving Recall).

As shown in the parameter count comparison in **Table 3**, YOLO series models have a parameter range of 1.97M~3.01M but lack sufficient accuracy; EfficientViT (4.01M) and FRPNet (4.65M) improve accuracy by increasing parameters yet are not conducive to edge-side adaptation. In contrast, IV-YOLO has a parameter count of only 2.52M, which falls within the parameter range of the YOLO series. Through the collaboration of the MSIV and GFEN modules, it achieves the dual advantages of “high accuracy and lightweight design,” making it suitable for UAV edge-side scenarios.

**Table 3:** The parameter comparison results.

Model	EfficientViT	Yolov8	Yolov9	Yolov10	FRPNet	Ours
Params (M)	4.01	3.01	1.97	2.26	4.65	2.52

**Figure 5:**
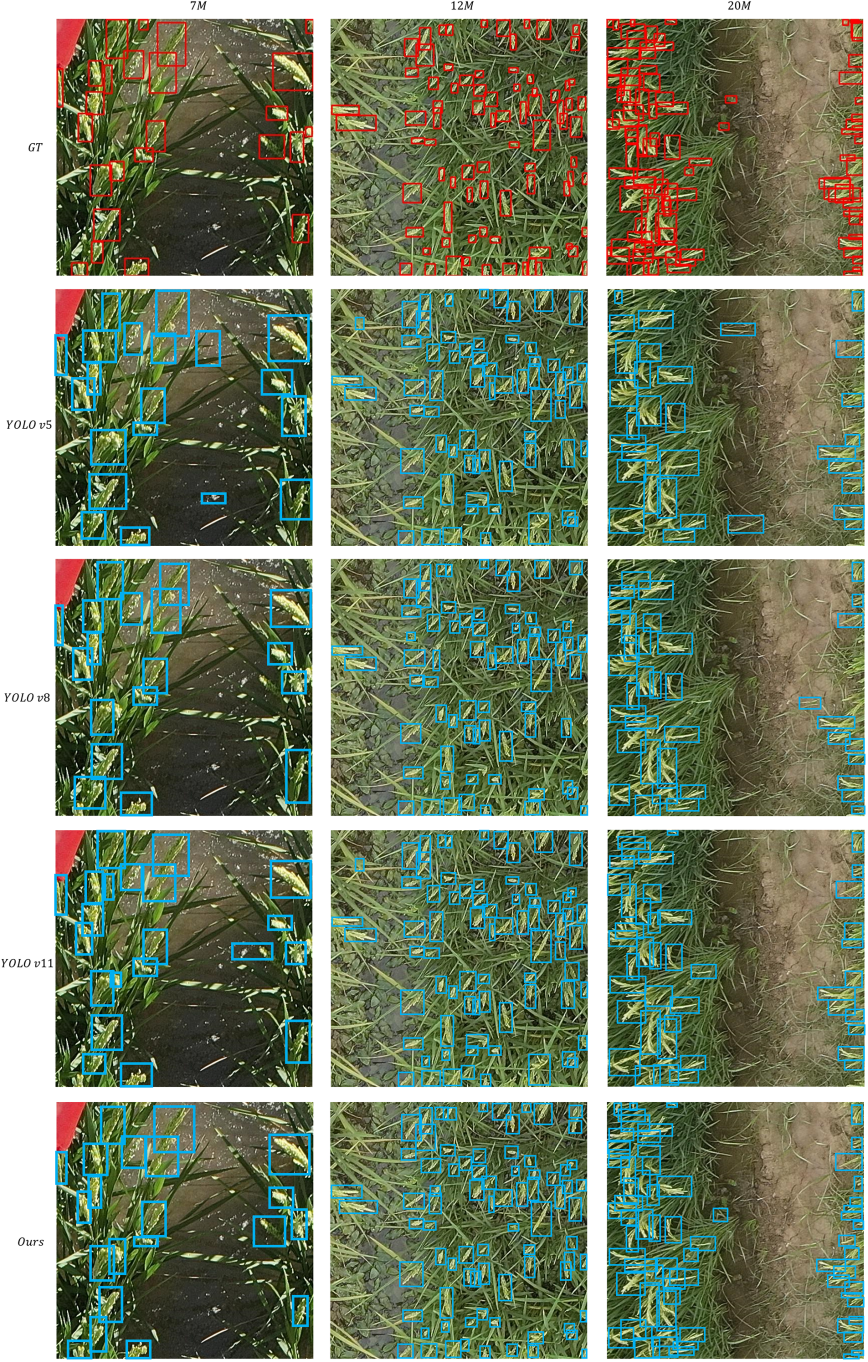
Qualitative comparison of experimental results.

**Figure 5** presents the qualitative comparative experimental results of different target detection models in crop scenarios. The first row labeled “GT” (Ground Truth) shows the bounding boxes of real targets (in red); the remaining rows sequentially display the detection results of YOLOv5, YOLOv8, YOLOv11, and the proposed “Ours” model (with bounding boxes in blue). Columns correspond to different scenario scales (7M, 12M, 20M).It can be observed that YOLOv5 exhibits numerous missed detections and localization deviations of bounding boxes across all scenarios. Although YOLOv8 and YOLOv11 show improvements, they still lack effectiveness in detecting small targets under dense or complex backgrounds. In contrast, the detection boxes of the proposed “Ours” model are more consistent with the ground truth annotations: missed detections and false detections are significantly reduced across different scenario scales, and targets are covered more accurately. This intuitively demonstrates the model’s superior target detection and localization capabilities, which aligns with the performance advantages reflected in the quantitative experiments.

### 4.3 Ablation experiment

Ablation experiment results demonstrate that the core components of the IV-YOLO model—namely the Multi-scale Information Vortex (MSIV) and Gradual Feature Fusion Neck (GFEN)—play a critical role in enhancing performance.

As shown in **Table 4**, after removing the MSIV module, the Recall decreases by 3.13%, the Average Precision (AP) decreases by 3.82%. This indicates the irreplaceable role of the MSIV in decoupling and enhancing fine-grained rice features (e.g., tiller edges, panicle textures).

**Table 4:** Results of the ablation experiment.

Model	Precision	Recall	AP	AP50
IV-YOLO	0.8596	0.8417	0.5569	0.8938
- MSIV	0.8476	0.8153	0.5356	0.8798
- GFEN	0.8028	0.8094	0.5327	0.8647

When the GFEN module is removed and replaced with another Neck (Khanam and Hussain, 2024) as the model’s Neck, the Precision decreases by 6.61%, the AP decreases by 4.35%, and the AP50 value decreases by 3.26%—verifying that gradual feature fusion is crucial for the semantic alignment capability of multi-scale rice targets (from tillers to panicles). GFEN adopts two-stage progressive fusion and embeds the MSIV module in each fusion step. It achieves progressive optimization of “feature decoupling - semantic alignment - feature refinement”. This not only addresses the feature coupling between rice plants and the background but also ensures accurate matching of shallow and deep features. In contrast, the alternative FPN only performs simple cross-scale feature concatenation. It lacks feature decoupling and anti-interference processing. This leads to confusion between rice plant features and clutter such as soil noise and weed textures. The false detection rate increases, resulting in a significant decrease in Precision.

Further analysis reveals a synergistic effect between the MSIV and GFEN modules. Through the progressive support of “feature decoupling → progressive fusion,” the two modules collectively ensure the high-precision detection performance of IV-YOLO in complex field scenarios (e.g., soil clutter, dense plant canopies).

## 5 Conclusion

To address the core bottlenecks of deep adhesion of rice plant features and field background clutter interference in rice detection using UAV remote sensing, this study proposes an Information Vortex-based Progressive Fusion YOLO (IV-YOLO) model to support the demand for rice phenotypic quantification in precision agriculture. Inspired by the water vortex mechanism in rice paddies, the Multi-scale Spiral Information Vortex (MSIV) module achieves decoupling of adhered rice plant features and suppression of background clutter via multi-scale rotational kernel convolution and channel-spatial joint reorganization. The Gradual Feature Fusion Neck (GFEN) balances shallow details and deep semantics, resolving the adaptability issue of traditional feature pyramids. Experiments based on the public DRPD dataset (5,372 RGB images, 259,498 annotated rice panicles, covering 4 growth stages and 3 remote sensing resolutions) demonstrate that IV-YOLO achieves a Precision of 0.8581, Recall of 0.8417, and AP of 0.5569—outperforming YOLO-series models and FRPNet across all metrics. In particular, its AP75 (0.6131) is 0.75 percentage points higher than that of FRPNet. Ablation experiments further confirm the necessity and synergistic effect of MSIV and GFEN. IV-YOLO provides a reliable solution for individual rice plant-level detection, supporting variable management and yield prediction in precision agriculture. Moreover, its “natural phenomenon inspired engineering design” approach offers a new paradigm for crop phenotypic analysis in agricultural remote sensing, facilitating the large-scale implementation of precision agriculture.

Although IV-YOLO has demonstrated excellent rice detection performance on public datasets, it still suffers from insufficient dataset generalization—its samples do not fully cover the topographies of different rice-growing areas (e.g., hills/plains), variety differences between indica and japonica rice, and extreme environments such as heavy rain and low light.

Future research can focus on constructing multi-source heterogeneous rice remote sensing datasets, incorporating samples from different regions, varieties, and extreme environments to enhance the model’s generalization ability.

## Data Availability

The original contributions presented in the study are included in the article/supplementary material. Further inquiries can be directed to the corresponding author.
